# Assessing the application of the revised Remane Model to fish species in a fluvially dominated cool-temperate southern African coastal system

**DOI:** 10.1038/s41598-023-31259-7

**Published:** 2023-05-08

**Authors:** Festus P. Nashima, Nadine A. Strydom, Stephen J. Lamberth

**Affiliations:** 1 Department of Fisheries and Ocean Sciences, University of Namibia, P.O Box 462, Henties Bay, Namibia; 2 Department of Zoology, Nelson Mandela University, P.O Box 77000, Port Elizabeth, South Africa; 3 Department of Environment, Forestry and Fisheries, Cape Town, South Africa; 4 Institute for Coastal and Marine Research, Ocean Sciences Campus, Nelson Mandela University, Gomery Avenue, P.O Box 77000, Port Elizabeth, 6031 South Africa

**Keywords:** Ecology, Zoology

## Abstract

Estuaries are ecologically important areas which support a variety of aquatic species, particularly marine and estuarine fish species. This study represents a case study of the Orange River and Estuary (ORE) to understand patterns in fish assemblages and diversity trends that were compared to the revised Remane Model predictions in an estuary with poor marine fish species richness. A total of 30 species were recorded in the River continuum that comprised 14 freshwater, 10 marine and six estuarine species. Species diversity revealed seasonal variation in fish assemblages during the high-flow and low-flow seasons but not inter-annually. The results revealed that species diversity is lower in higher salinity areas when compared to low salinity areas. These patterns are consistent with the biogeographical trend of decreasing species richness along the South African coast from east to west, however, inconsistent with Remane predictions. The extremely low marine fish richness at its lower end and the extremely large freshwater influx at its upper end are the primary causes of the inconsistency. This may reflects the unsuitability of the Remane model for the Orange Estuary. In comparison to similar river-dominated South African estuaries, the ORE has a low marine species richness. When compared to more conventional South African estuaries, the ORE has a unique biotic environment with low fish species richness of estuarine-associated marine species adjacent to the Benguela upwelling zone and therefore the region does not offer suitable habitat for such species. As a result, the ORE is not a good candidate to test the Remane Model. The data does, however, confirm the left-hand part of the Remane model, which shows a decline in the fish species richness of freshwater fish species as salinity rises towards mesohaline and polyhaline levels.

## Introduction

Estuaries constitute ecosystems of biological and ecological importance worldwide^1–4^. These ecosystems serve as breeding grounds for estuarine fish species, as well as nursery areas for juvenile fishes of marine origin^5,6^. Estuarine ecosystems are highly dynamic with steep environmental gradients, which influence the distribution of fish species depending on their tolerance ranges of abiotic and biotic variables, and the associated variability^7,8^. The Remane Model is one of the ecological models used to describe how aquatic faunal diversity changes along the transitional salinity gradient^9^, as well as the relative distribution of each component of freshwater, brackish-water and marine species^10,11^.

Fishes that utilize an estuarine environment exhibit a wide range of environmental tolerances and preferences as a result of adaptation to these highly variable environments^12^. However, several fish species may be subject to physiological limitations resulting in temporal changes in species composition, distribution and abundance^11,13,14^. In most South African estuaries including the Orange Estuary, temperature and salinity are the primary drivers structuring fish communities^15^. In contrast to the Berg and Olifants estuaries, the Orange Estuary is a unique system. It is the only South African estuary where freshwater fish species outnumber marine and estuarine fish species in terms of species richness, and it is the only one where fish species diversity is lower in high salinity areas and vice versa.

The Remane Model attempted to describe variation in fish species diversity along a salinity gradient typically associated with estuarine ecosystems and depicted this in diagrammatic form^16^. Moreover, the concept sought to comprehend the relative distribution of each component of freshwater, brackish-water and marine species along the salinity gradient^10,11^. However, the Model and associated diagram received criticism due to its several limitations^10,15,17–20^. Vannote et al.^21^ expanded the diagram by including fishes in an attempt to describe species distribution associated with salinity and synthesize a set of general hypotheses about communities along a river-estuarine gradient. Hedgpeth^18^, expanded the original diagram to include the entire salinity spectrum. Hudson^18^ then expanded on the shape of the original diagram by depicting the freshwater biota as having lower species diversity than the marine biota, as well as extending the presence of estuarine species beyond the 20 salinity limit. Whitfield et al.^11^ presented a revised model that demonstrated the connection between salinity and faunal trends in estuaries using fishes. Despite the modifications, it is generally acknowledged that the Remane Model remains hypothetical and still remains testable for fishes in estuarine ecosystems and their associated rivers^10^.

The ORE is a species-poor system situated in a broad area of the coast that is mostly a desert and falls within a cool temperate climatic region^5^. Moreover, the Orange Estuary is geographically isolated from other estuaries in the area and therefore devoid of any functional estuaries nearby. The estuary and river system were extensively sampled over a long-term period to assess the fish community dynamics along the river-estuary continuum. This data set afforded the researcher an opportunity to assess the applicability of the revised Remane Model to a river-dominated ecosystem that is unique compared to other South African estuaries in that freshwater fish species outnumber marine fish species and estuarine fish species, respectively, in terms of species richness. This study aimed to assess fish community structure, abundance and diversity along the Orange River and Estuary continuum and to test the applicability of the revised Remane Model to findings.

## Results

### Fish species composition

Over the eight-year study period, a total of 26,086 fish were caught, representing 30 species. According to the previous study by Nashima et al.^40^, the majority of fish species belonged to the freshwater category (47%) based on estuarine associations, with 11 freshwater stragglers and three freshwater estuarine-opportunist species recorded. The estuarine category (20%) included one solely estuarine species, five estuarine and marine species, and no estuarine and freshwater or estuarine migrant species. The marine category (34%) included four marine stragglers, three marine estuarine-opportunists, and three marine estuarine-dependents^40^.

### Seasonal and spatial trends in species diversity

**Figure 1:**
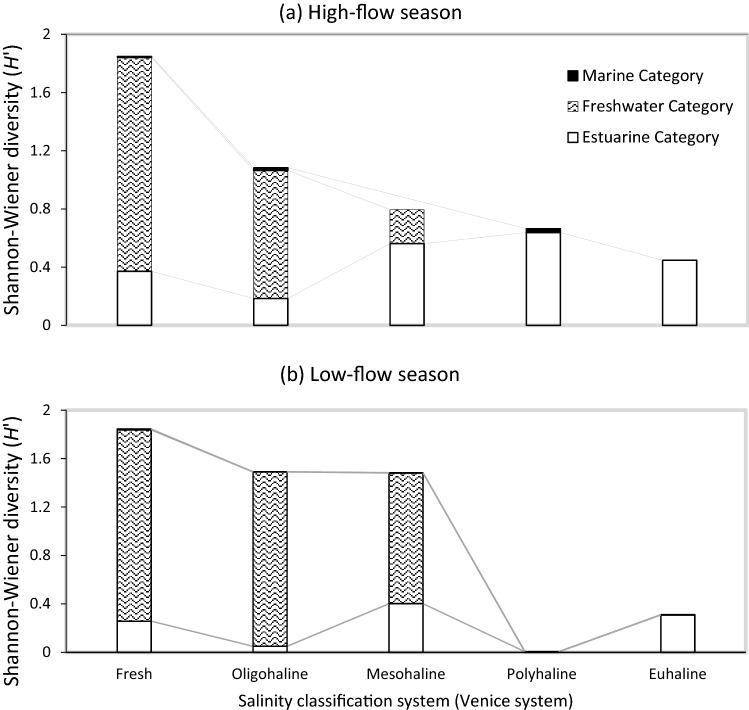
Seasonal changes in estuarine functional grouping covering the salinity continuum from freshwater to euhaline conditions based on an adaptation of the Venice system for the classification of salinity zones^39^.

The number of species (S) and Shannon–Wiener diversity (*H*') differed significantly between the high-flow and low-flow seasons (S: Mann–Whitney U-test, z = − 6.88, *P* < 0.001; *H*': Mann–Whitney U-test, z = − 5.01, *P* < 0.001). Both diversity indices depicted higher values during the high-flow season (Table 1). Species diversity indices showed significant differences among salinity zones recorded in both high-flow and low-flow season (*P* = 0.01, n = 201) but not years (*P* = 0.202, n = 8). Overall, both showed an increasing trend upstream into freshwater (Fig. 1; Table 1). Water temperature (z = − 10.69; *P* = 0.001) differed significantly in the high-flow period compared to the low-flow period. During the high flow period, the mean water temperature was 23.2 °C, with a range of 12.7–27.8 °C, and during the low flow period, it was 16.5 °C, with a range of 11.1–24.2 °C. During the high flow period, salinity averaged 4.1, with a range of 0.1 to 34.2, and during the low flow period, salinity averaged 7.5, with a range of 0.1 to 34.9. Whilst along the ORE, the study revealed significant differences (H = 25.30; d.f. = 5; *P* < 0.001) in water temperature which increased gradually upstream whilst salinity decreased sharply upstream.

**Table 1:** The diversity indices (S and *H*’) of fish assemblages recorded in different salinity zones during the high-flow and low-flow season in the Orange River Estuary during 2004–2018.

	No. of species (S)	Shannon–Wiener diversity (*H*')
Salinity zone (Range)		
Fresh (0–0.49)	24	1.05
Oligohaline (0.5–4.9)	23	0.41
Mesohaline (5.0–17.9)	14	0.13
Polyhaline (18.0–29.9)	10	0.05
Euhaline (30.0–35.9)	10	0.14
Season		
High-flow (*n* = 80)	28	0.60
Low-flow (*n* = 121)	27	0.27
Entire study area	30	3.56

### Estuarine species association along the river-estuarine continuum

This study recorded a salinity range of 0.1 to 34.9. The dominance of freshwater species in freshwater during the high-flow and low-flow seasons was followed by a rapid decline towards oligohaline and then more saline water (Fig. 1). Moreover, the majority of freshwater species were restricted to salinities below mesohaline conditions (5.0–17.9). During the high-flow season, *Tilapia sparrmanii* was the only freshwater species found in polyhaline water (2005 and 2007). During the low-flow season, no freshwater taxa were recorded in either polyhaline (18.0–29.9) or euhaline (30.0–35.9) waters (Table 2).

**Table 2:** Species checklist of presence ( +) and absence ( ) of fish species in varying salinity zones recorded in the Orange River Estuary during 2004–2018, based on estuarine usage functional guild.

Scientific name	EUFG	High-flow season					Low-flow season				
		F	O	M	P	E	F	O	M	P	E
*Gilchristella aestuaria*	Solely estuarine	+	+	+	+	+	+	+	+	+	+
*Caffrogobius nudiceps*	Estuarine & marine	+	+	+	+				+		+
*Caffrogobius saldanha*	Estuarine & marine	+	+	+	+	+		+	+	+	+
*Clinus superciliosus*	Estuarine & marine		+						+	+	+
*Atherina breviceps*	Estuarine & marine	+	+	+	+	+		+	+	+	+
*Syngnathus temminckii*	Estuarine & marine	+	+	+			+				+
*Chelon richardsonii*	Marine-estuarine-opportunist	+	+	+	+	+	+	+	+	+	+
*Lichia amia*	Marine-estuarine dependent		+								
*Pomatomus saltatrix*	Marine-estuarine-opportunist		+		+			+			
*Mugil cephalus*	Marine-estuarine dependent		+				+				
*Rhabdosargus globiceps*	Marine-estuarine-opportunist				+						+
*Lithognathus lithognathus*	Marine-estuarine dependent						+				
*Lithognathus aureti*	Marine straggler		+								
*Chelidonichthys capensis*	Marine straggler		+		+			+	+		+
*Cynoglossus capensis*	Marine straggler										+
*Austroglossus microlepis*	Marine straggler		+								
*Labeobarbus aeneus*	Freshwater straggler	+	+	+			+	+	+		
*Oreochromis mossambicus*	Freshwater estuarine-opportunist	+	+	+			+	+			
*Tilapia sparrmanii*	Freshwater straggler	+	+		+		+		+		
*Mesobola brevianalis*	Freshwater straggler	+					+	+	+		
*Pseudocrenilabrus philander*	Freshwater estuarine-opportunist	+	+				+	+			
*Labeobarbus kimberleyensis*	Freshwater straggler	+									
*Labeo umbratus*	Freshwater straggler	+					+				
*Clarias gariepinus*	Freshwater estuarine-opportunist	+					+	+			
*Cyprinus carpio*	Freshwater straggler	+	+				+	+			
*Barbus trimaculatus*	Freshwater straggler	+					+	+			
*Barbus hospes*	Freshwater straggler	+					+	+			
*Barbus paludinosus*	Freshwater straggler	+					+				
*Labeo capensis*	Freshwater straggler	+		+			+	+			
*Gambusia affinnis*	Freshwater straggler	+					+				

*F* fresh, *O* oligohaline, *M* mesohaline, *P* polyhaline, *E* euhaline.

The highest species diversity values in the marine category were recorded in oligohaline waters during a high-flow season (Table 1), with three marine estuarine-opportunists (*Chelon richardsonii, Pomatomus saltatrix*, and *Rhabdosargus globiceps*), three marine stragglers (*Lithognathus aureti, Austroglossus macrolepis*, and *Chelidonichthys capensis*), and two marine estuarine-dependent (*Lichia amia* and *Mugil cephalus*) species caught. The absence of marine estuarine-dependent species (*M. cephalus and Lithognathus lithognathus*) in salinities above oligohaline was notable (Table 2).

*Chelon richardsonii* and *M. cephalus* were the only two marine species with unrestricted distribution into freshwater reaches (Table 2). Other marine species, such as *L. amia, C. capensis, L. aureti*, and *P. saltatrix*, were only found up to the oligohaline zone (Table 2). Despite this, all other species, regardless of origin, were underrepresented in the Orange River Estuary continuum in terms of percentage occurrences, except for *C. richardsonii*^40^.

**Figure 2:**
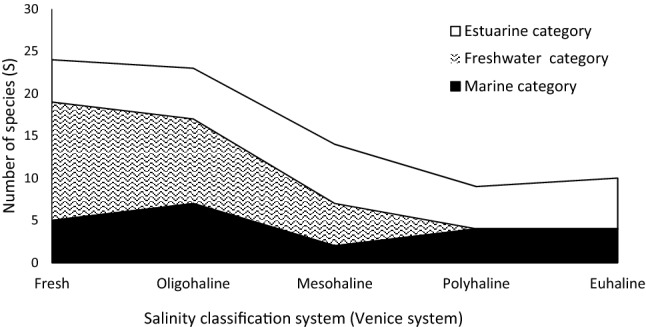
Trends in the number of species recorded during the entire study period 2004–2018, in the Orange River Estuary, showing changes in fish association along the salinity continuum based on an adaptation of the Venice system for the classification of South African salinity zones in estuaries^39^.

*Gilchristella aestuaria*, a solely estuarine species, and *C. richardsonii*, a marine estuarine-opportunist, were widely distributed across salinity zones. Overall, the number of species recorded in the ORE, showed the dominance of freshwater species in fresh and oligohaline waters while estuarine species dominate polyhaline and euhaline waters (Fig. 2).

## Discussion

The Remane Model was conceptualized in the Baltic Sea and widely used to explain estuarine biotic distributions^10,35,41^. Despite the fact that the model was not derived from estuary data and was solely based on invertebrates sampled in the brackish Baltic region, the Remane Model has been extended and modified over the years to provide an alternative generalized model for describing diversity trends in estuaries (e.g.^10,17–20^). Whitfield et al.^11^ presented a review and revised model for describing faunal biodiversity patterns along a salinity gradient in estuaries which included fishes. The revised model shows how the relative proportions of freshwater, estuarine and marine fishes change along a salinity gradient^11^. As a result, this study used the ORE continuum as a case study to determine whether the hypothesis presented by the revised Remane Model could be applied and tested using data on the ORE's longitudinal changes in fish diversity.

According to Whitfield et al.^11^ Remane revised model, it was revealed that freshwater taxa have fewer species than marine taxa in the same estuary; it was also stated that the vast majority of freshwater species are restricted to freshwater habitats, with only a few taxa extending into mesohaline, polyhaline, and euhaline zones. Moreover, marine taxa predominate in estuarine waters that are mesohaline, polyhaline, or hyperhaline. It was also revealed that although in small numbers, marine species can be found in oligohaline estuaries and even freshwaters. Estuarine species were found to be more diverse in mesohaline and polyhaline waters, but they can also be found in oligohaline, euhaline, and hyperhaline waters. Furthermore, a reduction in species diversity from high saline to low saline waters was observed, notably above a salinity of about 40.

The overwhelming dominance of freshwater fish species sampled in the riverine section of the ORE fully supports the revised Remane Model. The lower part of the Orange Estuary however was distinct in that it had low fish species diversity in comparison to other comparable river-dominated estuaries such as the subtropical Thukela and cool temperate Breede Estuary^42,43^. Moreover, the Berg and Olifants estuaries, also located in the cool-temperate bioregion of South Africa, has a good representation of estuary-associated marine fish species in the estuary and adjacent coastal zone^44,45^. The generally low species diversity of the ORE corresponded with the South African biogeographical trend of decreasing species richness from east to west^5^. The ORE is dominated by freshwater biota rather than typically marine and estuarine species, with species diversity declining with the increasing salinity continuum. Based on the estuarine association categories, the distribution of freshwater, estuarine and marine species differed significantly among salinity zones but did not differ among guilds.

The solely estuarine *G. aestuaria* was present among all salinity zones along the river-estuarine continuum. Freshwater stragglers dominated the freshwater zone and progressively declined from oligohaline water toward more saline water up to a salinity of 20.70 (polyhaline), while estuarine species progressed into euhaline water. Three *T. sparrmanii* were recorded in mesohaline/polyhaline water during the high-flow season. As expected, Tilapias are known to be tolerant of higher salinity stress^46^. Three species of freshwater estuarine-opportunists *Clarias gariepinus, Pseudocrenilabrus philander* and *Oreochromis mossambicus* were present in fresh and oligohaline water but the latter species penetrated further into the mesohaline zone but not into the polyhaline and euhaline reaches of the Orange Estuary. Mozambique tilapia *O. mossambicus* has been recorded in salinities ranging from 0 to 100^4,22^, despite its highly euryhaline characteristics, surprisingly it was absent in polyhaline and euhaline reaches of the Orange Estuary.

Estuarine and marine species were also abundant in euhaline water (i.e. *Syngnathus temminckii, Caffrogobius nudiceps, Caffrogobius saldanha, Clinus superciliosus* and *Atherina breviceps*) but this declined gradually toward polyhaline (10.0–29.9) waters and thereafter their abundance decreased in freshwater. The presence of marine estuarine-dependent species *M. cephalus, L. lithognathus* and *L. amia* in fresh and oligohaline water are not surprising as they are known to penetrate upriver into freshwater determined by salinity tolerance^5,22,45^. The presence of piscivorous predators such as *L. amia* and *P. saltatrix* can indicate feeding usage of the ORE continuum by the two species. Overall, the low marine fish species diversity in the Orange Estuary may be related to the high dominance of riverine influences throughout the estuary. However, *C. richardsonii* is dominant throughout the ORE continuum in terms of abundance and frequency^40^.

On the other hand, spatial variation in fish diversity along the salinity gradient can be influenced by spatial variation in other environmental parameters. Several authors have reported on the effects of environmental parameters on fish distribution and abundance in southern African estuaries^4,13^. Temperature and salinity are the primary drivers reported to structure fish communities in most South African estuaries^15^. In the current study, water temperature was significantly lower closer to the mouth of the estuary as a result of the ingress of cold upwelled seawater. The marine species *C. richardsonii* dominated there. Most fishes are ectothermic animals, therefore, their bodies do not produce heat to maintain constant and normal body temperature. Instead, they rely on the environment and their behaviour such as moving into more favourable areas to regulate their body temperature^47^. As a result, this and other environmental parameters may play a role in determining the distribution of fishes along the ORE continuum. The results of this study revealed that the ideas presented in the revised model^11^ do not fully reflect the diversity trends for fishes along the ORE continuum due to the exceptionally low fish species richness of estuary-associated marine taxa in the Benguela upwelling zone adjacent to the Orange Estuary mouth.

This study observed the following features related to fish diversity and each component of freshwater, brackish-water and marine species along the ORE: Both marine and estuarine species were present in all salinity zones (i.e. fresh, oligohaline, mesohaline, polyhaline, and euhaline waters); Freshwater species outnumber marine and estuarine species in terms of taxa; The majority of freshwater species were confined to freshwater, oligohaline, and mesohaline waters, with only a few taxa extending into polyhaline waters. No freshwater species were found in euhaline waters; estuarine species are more diverse in euhaline waters than in freshwater, oligohaline, mesohaline, and polyhaline waters; The ORE continuum is dominated by a single marine species (*C. richardsonii*). Moreover, a general decrease in species diversity was observed as one moves from low to high salinity waters.

In comparison to the revised models^11^, it was observed that fish assemblages that utilize the river-estuarine environment, particularly the dominant species, use a wide salinity range (i.e. plasticity) along the ORE (e.g. *C. richardsonii* and *G. aestuaria*). Overall, species diversity increased upstream in the river due to the dominance of freshwater fishes coupled with several marine and estuarine species. Freshwater fish species predominate in the ORE, however there are few marine species that are connected with estuaries. This is exclusive to the ORE since no other estuary in Southern Africa have the same qualities. Such patterns are brought about by the fact that Orange is a lone system on a huge stretch of coast that is mostly desert and devoid of any nearby functioning estuaries. Additionally, the system is unsuitable for certain species, and variations in species tolerance and preference for changes in salinity throughout the ORE can determine which fish species are present along the river system. As a result, this rendered Orange Estuary unsuitable as a testing ground for the Remane model.

In conclusion, this study used the ORE continuum as a case study to describe the longitudinal distribution of fish species in relation to a full salinity gradient and compare this with hypotheses based on the revised Remane Model. This study confirmed the dominance of freshwater taxa in the freshwater/oligohaline zone of the estuary as per the revised Remane Model. However, the decline in species diversity/richness in the higher salinity waters was attributed to the lack of estuarine–associated marine fish species available to recruit into the ORE from cold coastal waters situated in an upwelling zone. Therefore, the revised Remane Model hypotheses were not supported in the ORE continuum highlighting the need to assess estuaries and their dynamics on a case by case basis rather than attempting to secure a generic model of distribution patterns. More research is needed on applying existing ecological models to other river-dominated estuaries worldwide.

## Methods

### Study area

**Figure 3:**
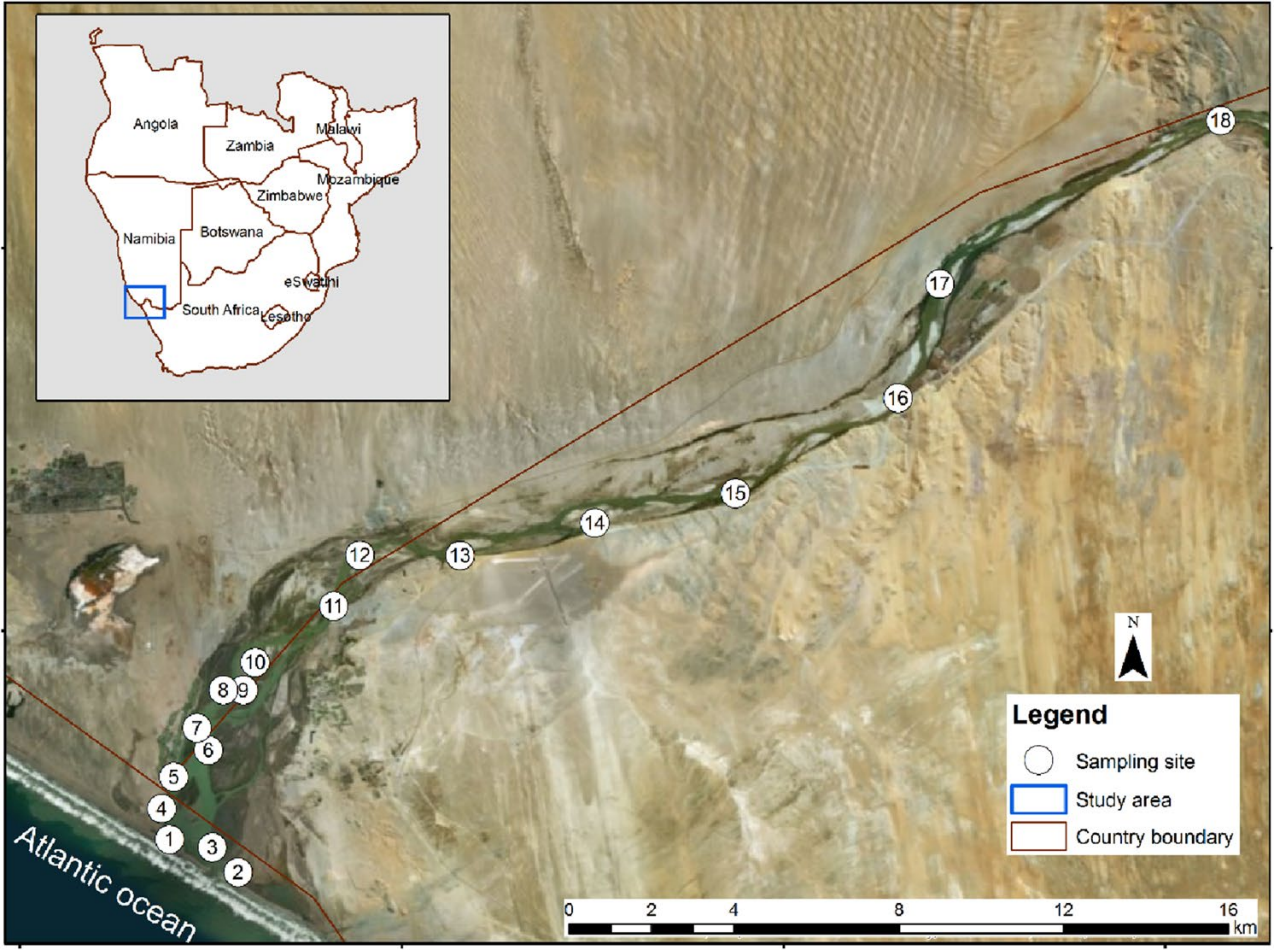
The Orange River Estuary is shown on the map from site 1 to 11, while the river section is shown from site 12 to 18. The image was obtained from Google Earth Image and was used as a base map in ArcGIS to create the map^24,25^.

The Orange River flows over 2300 km from its origin in the highlands of Lesotho downstream into the Atlantic Ocean on the northwest coast of South Africa^22,23^. At the mouth, there is an estuary that forms the border between South Africa and Namibia. The Orange Estuary (28°35ʹ S, 16°30 ʹ E; Fig. 3)^24,25^ stretches from the mouth to about 10 km upstream^26^. Its catchment area covers 549,000 km^2^ and drains approximately 60% of South Africa's land area, while the remainder falls within Botswana (11%), Namibia (25%), and Lesotho (4%)^27^.

The Orange River Catchment falls within the summer rainfall zone whereas the Orange Estuary is located in an arid, cold temperate climate region^28^ and falls within a winter rainfall zone, where infrequent rainfalls average about 50 mm per year^22,29^. As a result, high-flow downstream occurs during the summer months rather than during the winter rainfall season^22^.

### Field sampling

Fieldwork was carried out during the typical high-flow (December-March) and low-flow (August-October) seasons^30^. Within a 15-year period, sampling was carried out in eight different years (2004, 2005, 2012, 2013, 2015, 2016, 2017 and 2018). Two sampling trips were conducted biannually for all years except 2012, 2013, and 2016 during the high-flow period, and also in 2017 during the low-flow period. Only four sites were not sampled due to low water levels or inaccessibility to the shore due to reed over-growth (sites 17 (2012) and 16 (2017) during high-flow season; site 13 (2017) and 16 (2018) during low-flow season). During any of the sampling trips, however, no very high flow was observed. This was due to river system regulation upstream by the Gariep and Vanderkloof dams which significantly reduced the amount of water flowing downstream to the estuary^31,32^. The two dams are located in South Africa. The Vanderkloof Dam is located approximately 130 km downstream of Gariep Dam and is fed by the Orange (or Gariep) River, South Africa's largest river. Vanderkloof Dam is South Africa's second-largest dam, with the highest dam wall in the country standing at 108 m. Prior to the construction of these dams during the late 1960s and early 1970s, the Orange River displayed a distinct seasonal flow pattern, with high-flow during summer months and low-flow periods during winter. Subsequently, impoundments of summer high-flow and hydropower releases during winter low-flow has often resulted in “seasonal reversals” inflows^31,32^. Throughout this study, the Orange Estuary remained open (2004–2018).

A beach-seine (30 × 2 × 10 m) was used for sampling at 18 sites from the mouth of the estuary to 35 km upstream in the river (Fig. 3). The mesh size of the net was 10 mm in the centre, including a cod-end (bag), and 10 m of 15 mm stretched mesh size in each of the wings. The distances between sites varied greatly, and they were chosen to reflect a progression from marine to estuarine to freshwater influences as well as accessibility to the water course. Fish caught were identified to species level^33,34^, measured and counted at each site. After recording, fishes were released alive into the water where possible. All applicable guidelines for the care, collection and use of animals were followed in accordance with the ethical standards of Nelson Mandela University (NMU, South Africa). Specifically, the ethics clearance was granted by the Research Ethics Committee (Animal), Nelson Mandela University. The ethics clearance reference number is A18-SCI-ZOO-002.

The number of fish species that were collected per seine haul at each site was recorded. In situ temperature, and salinity (expressed as practical salinity units) were measured at each site, from the middle of the water column, using a Yellow Spring Instrument *v6920/EXO* 1 multi-parameter probe. The river and estuarine area were divided into salinity zones based on an adaptation of the Venice system of South African salinity zones in estuaries (freshwater: 0–0.49 PSU; oligohaline: 0.5–4.9 PSU; mesohaline: 5.0–17.9 PSU; polyhaline: 18.0–29.9 PSU; euryhaline: 30.0–35.9 PSU; and hypersaline: ≥ 36 PSU)^35^.

Moreover, fishes recorded were grouped into estuarine guilds following Potter et al.^36^. The marine category is divided into three guilds (marine straggler, marine estuarine-opportunist, and marine estuarine-dependent), the estuarine category is divided into four guilds (solely estuarine, estuarine & marine, estuarine & freshwater, and estuarine migrant), the diadromous category is divided into five guilds (anadromous, semi-anadromous, catadromous, semi-catadromous and amphidromous), freshwater category consists of two guilds, namely the freshwater straggler and freshwater estuarine-opportunistic^36^.

### Data analyses

The diversity indices including Shannon–Wiener diversity (*H*') and the number of species (S)) were calculated using PRIMER v6 statistical software package among flow season, years, and sites^37^. The assumptions of normality and homogeneity of variance were tested for salinity, temperature and diversity indices using a normal probability plot and Levene’s Test. All environmental data and diversity indices data revealed violation of both normality and homogeneity of variances and non-parametric tests were used.

Mann–Whitney *U*-test^38^ was used to test whether diversity indices (*H’* and S) differed between high-flow and low-flow season. Kruskal–Wallis tests were performed separately on species diversity indices to test for differences between years and salinity zones. Stacked area diagrams were created in Microsoft Excel 2013 to show the distribution of estuarine associations based on Potter et al.^36^, as well as a salinity continuum based on the revised Venice system for salinity zone classification^39^. Moreover, Kruskal Wallis test was performed to assess whether temperature differs between flow, season and location along the ORE. The significance level for all tests was set at *P* ≤ 0.05.

### Ethics declarations

All applicable guidelines for the care, collection and use of animals were followed in accordance with the ethical standards of Nelson Mandela University (NMU, South Africa).

### Approval for animal experiments

The ethics clearance was granted by the Research Ethics Committee (Animal), Nelson Mandela University, South Africa. The ethics clearance reference number is A18-SCI-ZOO-002.
